# LAP-Hsp60 complex modulates epithelial tight junction barrier

**DOI:** 10.21203/rs.3.rs-6474377/v1

**Published:** 2025-05-14

**Authors:** Manalee Samaddar, Chen Sun, Nicholas L.F. Gallina, Nicole Irizarry-Tardi, Dongqi Liu, Shivendra Tenguria, Rishi Drolia, Anika Jain, Daisuke Kihara, Wen Jiang, Kee-Hong Kim, Abigail Cox, Kurt D. Ristroph, Gregory T. Knipp, Nicholas Noinaj, Arun K. Bhunia

**Affiliations:** 1 Molecular Food Microbiology Laboratory, Department of Food Science, Purdue University, West Lafayette, IN, USA; 2 Purdue Institute of Inflammation, Immunology, and Infectious Disease, Purdue University, West Lafayette, IN, USA; 3 Department of Biological Science, Purdue University, West Lafayette, IN, USA; 4 Department of Agricultural and Biological Engineering, Purdue University, West Lafayette, IN, USA; 5 Department of Biological Science, Old Dominion University, Norfolk, VA, USA; 6 Department of Food Science, Purdue University, West Lafayette, IN, USA; 7 Department of Comparative Pathobiology, Purdue University, West Lafayette, IN, USA; 8 Davidson School of Chemical Engineering (by courtesy), Purdue University, West Lafayette, IN, USA; 9 Department of Industrial and Physical Pharmacy, Purdue University, West Lafayette, IN, USA

**Keywords:** LAP, Hsp60, cryo-EM, structure-function, peptide drug delivery, mouse, tight junction modulator

## Abstract

During infection, *Listeria* adhesion protein (LAP), a housekeeping enzyme, acts as a tight junction modulator (TJM) through interaction with Hsp60 to facilitate *Listeria monocytogenes* translocation across the intestinal epithelial barrier. Here, we used purified LAP as a potential TJM to overcome the limiting and variable effects observed by other agents in the class. We structurally determined the LAP interaction alone and in complex with Hsp60 utilizing cryo-EM and computational analysis. LAP structure resolved at 2.83 Å, forms multimeric interlocking dimers and tetramers, and the N-domain interacts with Hsp60, while the C-domain bridges bacterial surface receptor InlB. The structural studies complement LAP-mediated cyclic peptide drugs (vancomycin and desmopressin) absorption across the intestinal barrier in a mouse model without inducing inflammation or adverse effects on the TJ architecture. This study demonstrates that the LAP-Hsp60 complex is the basis for downstream utilization of LAP or peptide mimetics as promising TJM for improved peroral biologics delivery.

## Graphical Abstract

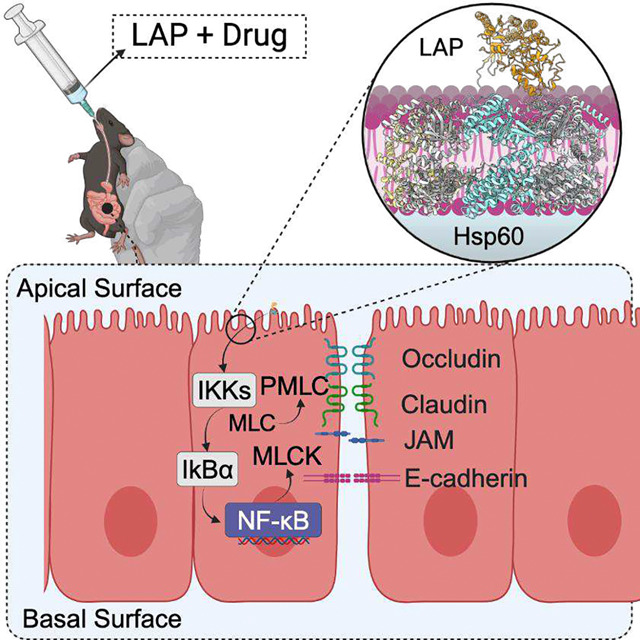

## Main

Oral drug administration of biological-based therapeutics (‘biopharmaceuticals’ or ‘biologics’) presents a significant challenge for pharmaceutical scientists, particularly as the number of peptide-based therapies continues to increase^1^. Oral delivery offers convenience for patient compliance and shows promise in stable, long-lasting formulations^2^ and controlled release mechanisms^3,4^. Despite these advantages, the gastrointestinal tract remains a formidable obstacle to efficiently delivering biopharmaceuticals^5^. The physical and biochemical properties of the intestinal epithelium have been a major obstacle in reducing the bioavailability of protein-based drugs^6^, often resulting in suboptimal absorption^7^, premature degradation^8^, and increased susceptibility to side effects^9,10^.

Strategies to improve peptide and protein luminal metabolic stability have been developed, such as cyclization^11^ and the advent of stapling peptides and proteins^12–14^. Advancements have also been made in designing drug formulations using e.g., nanoparticles and prodrugs to improve stability and epithelial transport^15,16^. But, the tight junctions between epithelial cells remain a major physical challenge preventing the efficient absorption of macromolecular biopharmaceuticals.

In the small intestine, epithelial tight junctions (TJ), comprised of specialized proteins, regulate the flow of molecules and ions between adjacent cells^17^. Several factors modulate a drug’s permeability across the intestinal epithelial layer and subsequent absorption^18^. Effective oral delivery remains elusive because many drug compounds are too large, hydrophilic, or hydrophobic to penetrate these restrictive TJs^19^.

The use of perturbants to increase the pore radius in epithelial tight junctions has long been explored to improve the peroral delivery of peptides and proteins but has met with little clinical success in part due to the instability of the molecules and transcience in the excipient effect^20–22^. Excipients, including acylcarnitines, have been tested in late-stage clinical trials and have revealed moderate success^23,24^, highlighting a need to develop novel/optimized perturbant molecules.

Several pathways exist for transporting substances across epithelial barriers. These are categorized as passive methods, such as paracellular and transcellular diffusion, energy-independent facilitated transport, and active mechanisms, such as endocytosis and transporter-mediated translocation^20,25^. Although the epithelial barrier is dynamic, allowing selective permeability of smaller molecules, the flux of larger molecules remains limited. Targeting TJs and adherens junctions (AJs) via newly-developed modulators has shown potential^26^, but with limited success due to their non-specific actions^27^ and variable absorption rates that lead to difficulties in maintaining a target plasma concentration for drugs with a narrow therapeutic index ^28^. These inefficiencies add a significant economic constraint to drug development and, ultimately, to patients^29^. Thus, developing TJ-modulating agents that can increase oral absorption in a controlled and reproducible manner represents an unmet area of need for the field.

During infection, enteric pathogens such as *Listeria monocytogenes* (Lm) have skillfully evolved mechanisms to open the intestinal barrier transiently^30,31^, thereby enabling structural alteration to the intestinal lining for systemic spread^32,33^. Specifically, Lm employs a targeted approach not just to breach the intestinal barrier but also to cross the blood-brain and placental barriers, underscoring its potency in overcoming host defenses^34^. The initial and perhaps most critical step in Lm’s infection cycle involves its ability to traverse the intestinal epithelial wall^33,35^ and serve as a gateway into the host’s internal systems, facilitating its further spread into deeper tissues^34,36^. In short, Lm’s adeptness at destabilizing the TJs of the intestinal barrier is a pivotal factor in its survival strategy, enabling it to adapt to and exploit the host environment for its propagation.

Lm employs the *Listeria* adhesion protein (LAP), a 94 kDa alcohol acetaldehyde dehydrogenase (AdhE, *lmo1634*), a housekeeping moonlighting enzyme^37^ to adhere to and destabilize host epithelial barriers, facilitating its invasion into subepithelial compartments^30^. LAP is a bifunctional enzyme (866 aa) consisting of N-terminal acetaldehyde dehydrogenase (ALDH; aa 1–452) and C-terminal alcohol dehydrogenase (ADH; aa 458–866)^38^, secreted using SecA2 machinery^39^ and anchored to the Lm cell surface via electrostatic interaction with the glycine (G) tryptophan (W) domain of internalin B^37,40^. Unlike genes within the *Listeria* pathogenic island 1, the *lap* gene is not regulated by the global virulence factors PrfA or σB^39^.

As an adhesion protein^38,41^, LAP interacts with the host cell receptor, human Hsp60^42^. Interestingly, secreted and membrane-bound Hsp60 is considered an immune messenger^43^ and a biomarker for metastatic cancer^44^, cardiovascular disease^45^, and inflammatory bowel disease^46^. The N2 subdomain of LAP has the strongest affinity (K_D_ = 9.50 ± 2.6 nM) for Hsp60 and is critical for host-cell interaction^47^. LAP activates canonical NF-κB signaling and myosin light chain kinase (MLCK) to destabilize junctional proteins to allow Lm crossing of the intestinal epithelial barrier before dissemination to deeper tissues^30,33^. Activation of the LAP-Hsp60-NF-κB-MLCK axis is instrumental in the transient opening of the junctional barrier via cytoskeletal contraction of claudin 1, occludin, and E-cadherin^30,33^ and caveolae-mediated (caveolin-1) apical junctional protein endocytosis^31^.

This study explores the potential of LAP as a TJM to boost oral peptide drug delivery, especially drugs with low bioavailability. LAP’s structure revealed a large, interlocking oligomeric spirosome assembly, where the N terminus of LAP interacts with Hsp60. The structural analysis further revealed peptide motifs that interact with Hsp60, which may further enable the design of smaller mimetics that can function analogously. LAP and smaller synthetic peptides notably increased epithelial permeability in cultured cell models and outperformed other established permeability enhancers at lower doses. In a mouse model, LAP improved intestinal barrier permeability within 2–4 h and elevated peptide drug levels in mice plasma. LAP induced a transient mislocalization of structural proteins for opening the tight junction barrier, which soon reverted to its original state without showing commensal bacterial translocation or inflammatory responses. These data validate LAP or its peptide mimetics potential utility as a tight junction modulator for effective and safe oral drug delivery of therapeutic peptides and proteins^48^.

## Results

### The cryo-EM structure of LAP

LAP was cloned into the pHIS2-Parallel vector, expressed in BL21(DE3) cells (**Supplementary Fig. 1 a-c**), and purified using nickel affinity chromatography and size-exclusion chromatography (SEC) using a Superdex 200 Increase 10/300 GL column (**Supplementary Fig. 1d**). The early elution peak by SEC indicated the formation of a large oligomer, which was confirmed by observing large distinct spirosome-like structures using negative-stain TEM (**Supplementary Fig. 1e**). Cryo-EM grids of LAP were then prepared and screened using an FC200 Talos electron microscope, with final datasets collected on a Titan Krios equipped with a Gatan K3 detector. A total of 2,239 micrographs were collected, with 2.3 million particles picked initially and 1.5 million subsequently selected for iterative 2D classification and subsequent homogeneous and non-uniform refinement in CryoSPARC (**Supplementary Fig. 1f-h**). The final LAP structure was resolved to 2.83 Å resolution from 1.2 million particles (**Supplementary Table 1**).

**Fig. 1 |:**
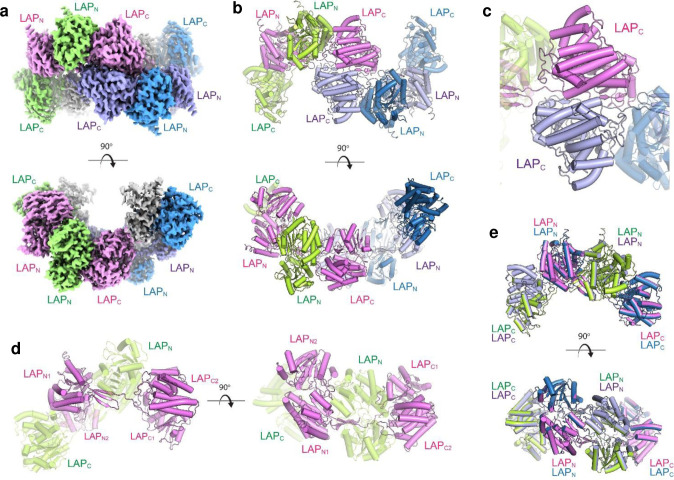
The cryo-EM structure of tetrameric LAP. **a.** The cryo-EM map of the LAP tetramer allows unambiguous assignment of the side chains. Each monomer is in a different color, and each domain is indicated. **b.** The tetrameric structure of LAP consists of interlocking subunits flanked on each end by C-domains. **c.** The tetramer consists of two dimers arranged in a pseudo-2-fold symmetry that assemble along the C-domain of LAP. **d.** Each dimer consists of extended interlocking monomers (green and pink), each consisting of an N-domain, an extended linker region, and a C-domain. **e.** Each tetramer structure consists of two LAP dimers (green/pink; blue/violet) that align well with one another with RMSD of 0.1 Å.

LAP was observed in dimeric, tetrameric, and large oligomeric forms, suggesting an equilibrium between these forms in solution (Fig. 1a,b and **Supplementary Movie 1**). Each tetramer, arranged in a spirosome-like assembly, was flanked on each end by an additional copy of the C-domain (ADH) of LAP; no density was observed for the corresponding N-domain (ALDH), indicating they were either wholly disordered or had been enzymatically removed. The core tetrameric structure consisted of a dimer of dimers with pseudo-2-fold symmetry, with the interaction between the dimers mediated solely by the interfacial C-domains (Fig. 1c). The dimers align well with one another with a root mean square deviation (RMSD) of 0.1 Å. Each dimer consists of interlocking monomers in a quasi-domain-swap assembly (Fig. 1d,e), with each monomer consisting of an N-domain, an extended linker, and a C-domain.

### The interaction of LAP with Hsp60 is mediated by the N-domain of LAP

**Fig. 2 |:**
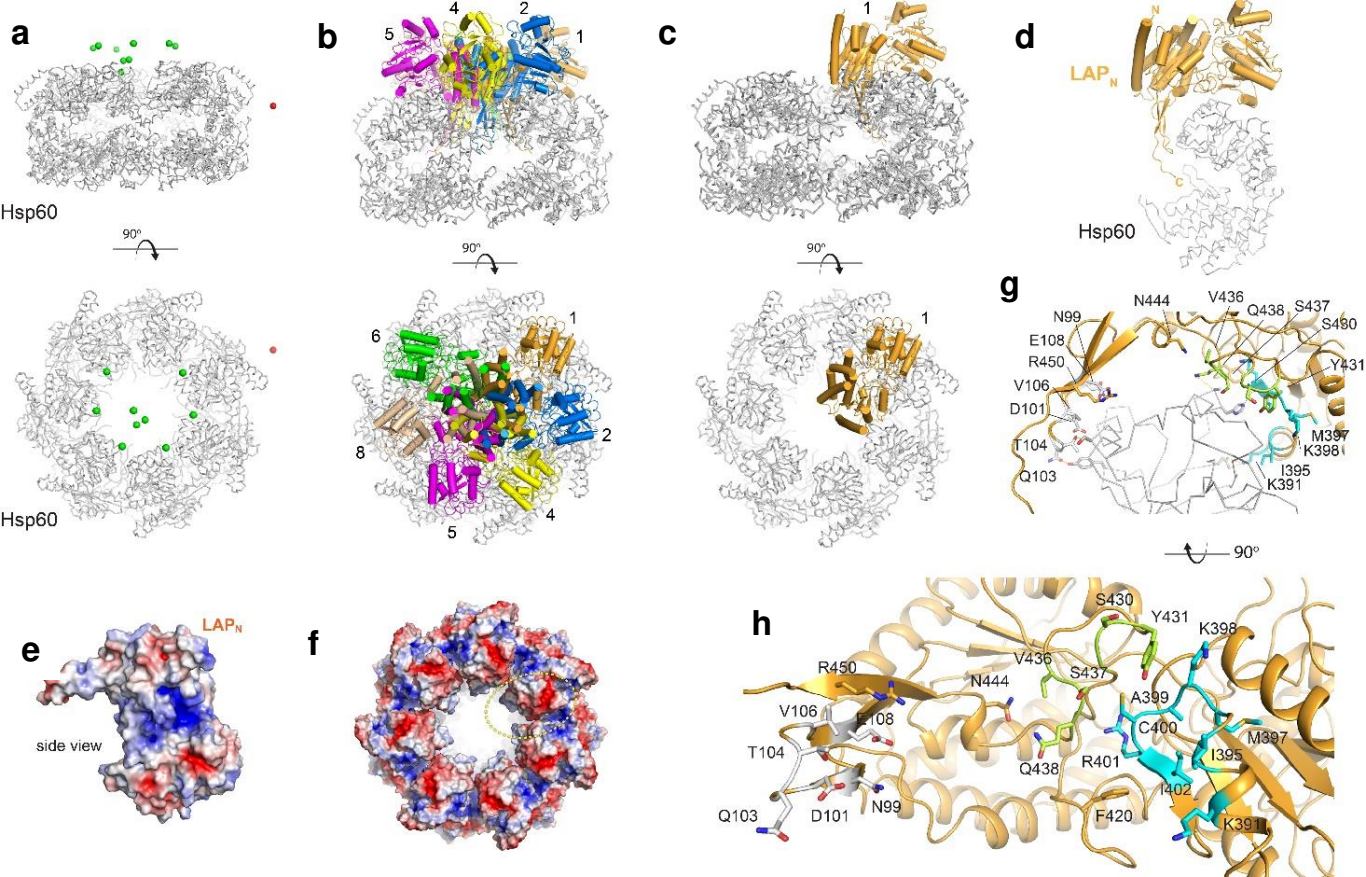
The structure of the LAP-Hsp60 complex using cryo-EM and computational modeling. **a.** The location of the top 10 solutions from the computational docking of the N-domain of LAP to Hsp60 (gray) are shown as spheres representing the center of mass of each solution; green agrees with the location found in our cryo-EM, red is in other locations. Orthogonal views are shown. **b.** The models are shown for each of the six solutions that agree with the cryo-EM and share similar binding modes, given that Hsp60 is a heptamer. **c.** Only the top solution is now shown of the N-domain of LAP (orange) with Hsp60 (gray). **d.** An enlarged view of only the N-domain of LAP (orange) with a monomer of the primary interaction molecule from Hsp60 (gray), depicting shape complementarity. **e.** The electrostatic properties of the N-domain of LAP show a strongly positive region (blue) along the region that interacts with Hsp60. **f.** The electrostatic properties of the Hsp60 assembly show strongly negative regions (red) at the interface of each of the Hsp60 monomers, where the N-domain of LAP binds (yellow dashed circle). **g.** An enlarged view of the interacting region of N-domain LAP (orange) with Hsp60 (gray). **h.** An orthogonal view of panel G looking at the binding interface of the N-domain of LAP consisting of a buried surface area of ~1000 Å^2^, with the interacting residues highlighted into the three primary interacting groups including (i) D101-E108 (gray), (ii) S430-Q438 (green), and (iii) K391-I402 (cyan).

Cryo-EM was used to study the structure of the complex to determine the basis for the interaction of LAP with Hsp60. Purified LAP was mixed with Hsp60 (**Supplementary Fig. 2a**) at a 2:1 molar ratio and examined using a Titan Krios equipped with a Gatan K3 detector. A total of ~4,500 micrographs were analyzed, with ~2 million particles picked initially and 200K subsequently selected for iterative 2D classification and subsequent homogeneous and non-uniform refinement in CryoSPARC (**Supplementary Fig. 2b**). Three distinct 3D classes representing tetrameric LAP, dimeric LAP, and Hsp60 were easily observed; no classes were observed representing dimeric or tetrameric LAP bound to Hsp60 (**Supplementary Fig. 2c,d,e**). Additionally, no obvious classes represented larger extended oligomers, as observed in the LAP-only dataset. While the dimeric and tetrameric LAP structures were consistent with the data for LAP alone, the maps for Hsp60 (refined in C1 symmetry) contained additional poorly-defined density that was consistent with the size and shape of the N-domain of LAP (**Supplementary Fig. 2e,f**). To further support this observation, HDOCK^49,50^ was used to model the interaction of the N-domain of LAP with Hsp60, which resulted in 9 of the 10 solutions showing the N-domain of LAP being positioned in the exact location on Hsp60 as modeled in our cryo-EM analysis (Fig. 2a, **Supplementary Movie 2**). Six solutions share almost identical binding modes, given that Hsp60 is a heptamer (Fig. 2b). For the top solution, the binding interface has a buried surface area of ~1000 Å^2^, which shares shape and charge complementarity (Fig. 2c,d and **Supplementary Fig. 3a,b**). This interacting interface is consistent with complementary electrostatic properties of the two proteins (Fig. 2e,f) and includes residues from three primary interfaces of LAP, including (i) D101-E108, (ii) S430-Q438, and (iii) K391-I402 (Fig. 2g,h). The residues at this interface from Hsp60 include D215, E236, R242, K243, E309, D310, H314, and K335 (**Supplementary Fig.3c** and **Supplementary Table 2**).

**Fig. 3 |:**
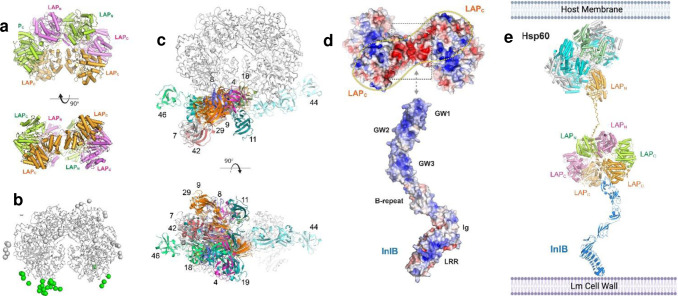
The cryo-EM structure of dimeric LAP and a structural model for the InlB-LAP-Hsp60 complex. **a.** A separate 3D class showed the dimeric structure of LAP, flanked on each end by C-domains; no density was observed for the N-domain for these flanking domains. **b.** AlphaFold was used to model the GW1–3 domains of lnlB with the LAP dimer (ribbon). The locations of InlB for the top 50 results are shown as spheres, with the green spheres clustered in proximity to the predicted interaction site indicated in the panel. **c.** Orthogonal views of the AlphaFold models are shown in cartoon representation for select InlB models indicated in green from panel **B**; the numbers indicate the rank of each model from AlphaFold. **d**. The electrostatic properties of InlB and the LAP dimer are shown with the indicated charge complementary surfaces. The dimeric interface in LAP across the C-domains is indicated by the yellow dashed oval, with the putative interacting interface displayed by the dashed black box. **e**. A model for the InlB-LAP-Hsp60 interaction complex is shown based on cryo-EM studies, analysis of surface electrostatics, and computational modeling.

Recently, we showed that secreted LAP anchors with the glycine (G)-tryptophan (W) domain of Internalin B (InlB) for surface localization on Lm^37^. To determine an improved model for the interaction of LAP with InlB, AlphaFold^49^ and LZerD^51^ were used to model the interaction of the GW repeats of InlB with the LAP dimer, representing the interaction during surface anchoring (Fig. 3a). Of the top 50 solutions, half were randomly scattered across the surface of the LAP structure with no obvious preference. The other half, however, was observed with the GW domains near the junction site where the additional C-domains of the dimer interact with pseudo-2-fold symmetry (Fig. 3b,c). These primary interacting interfaces have complementary electrostatic surfaces, where the electropositive GW repeats were found nested along the electronegative flanking C-domains of the LAP dimer (Fig. 3d).

Using our cryo-EM analysis of the LAP-Hsp60 complex, along with our computational model of LAP-InlB using AlphaFold, and supported by previous binding studies from our lab, we formed a complete model for the fully assembled InlB-LAP-Hsp60 intercellular adhesion complex spanning the Lm surface to the host membrane (Fig. 3e and **Supplementary Movie 3**). In our model, LAP is anchored to the *Listeria* cell surface with InlB via interactions with the flanking C-domains of the LAP dimer. At the same time, the N-domain of the LAP monomer is extended to interact with Hsp60 on the host membrane directly. Here, we postulate that while the C-domain of LAP is attached to InlB^37,40^, the N-terminal domain physically detaches from the LAP molecule and binds to Hsp60 in the host cell membrane. The model for this InlB-LAP-Hsp60 complex offers a complete working structural model of the multi-component assembly, laying a foundation for deeper insights into the bacterial adhesion process and its ramifications in pathogenesis^30,37^.

### LAP increases epithelial permeability through the cultured cell monolayers

**Fig. 4 |:**
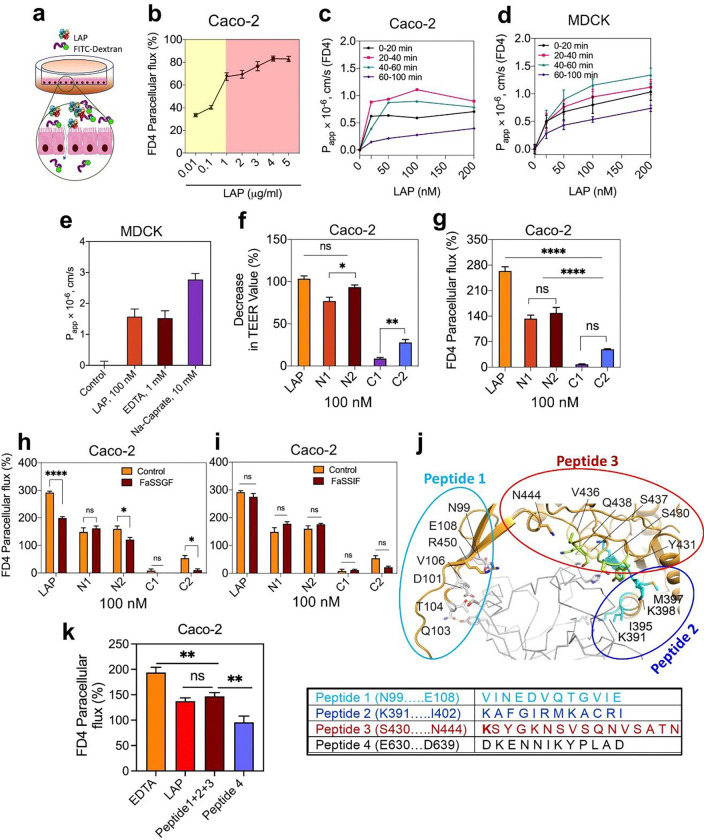
LAP increases paracellular permeability in cultured cell models. **a.** Overview of paracellular permeability study in transwell setup. **b.** LAP dose-dependent effect on FITC- 4 kDa dextran (FD4) permeability through Caco-2 cell monolayers. **c-d.** Apparent permeability coefficient ($P_{app}$) measurement of LAP-mediated FD4 through Caco-2 (c) and MDCK cell line (d). **e.** Comparison of $P_{app}$ value of FD4 after treatment with LAP, EDTA, and Na-Caprate. **f-g.** LAP and subdomain protein (N1, N2, C1, and C2)-mediated TEER and FD4 permeability through Caco-2 monolayers in 1 h. **h-i.** Effect of fasted simulated stomach (FaSSGF) and small intestinal (FaSSIF) fluid on LAP mediated FD4 permeability. **j-k.** Peptides from the N-terminal domain of LAP that interact with Hsp60 (j) and their effect on FD4 flux through Caco-2 monolayers in 1 h (k). Peptides 1, 2 and 3 were combined. Peptide 4 is located outside the Hsp60 binding domain. All error bars represent SEM (n = 3–6). ****p < 0.0001; **p < 0.01; *p < 0.05; ns, no significance.

We used purified endotoxin-free LAP and its subdomain proteins (N1, N2, C1 and C2) (0.01 – 5 μg/mL) (**Supplementary Fig. 5a-c**) as a TJM to assess their potential to deliver a model drug, fluorescein isothiocyanate (FITC)-labeled 4-kDa dextran (FD4) via the paracellular route exclusive of bacterial involvement in a transwell set up using Caco-2^52^ and Madin-Darby Canine Kidney (MDCK)^53^ cell lines. LAP-induced FD4 permeability in the Caco-2 cell monolayer, measured after one hour, was dose-dependent, peaking the permeability to about 67% (1 μg/mL; 10 nM) and then plateauing around 80% at and above a dose of 4 μg/ml (Fig. 4a,b).

We also measured the apparent permeability coefficient ($P_{app}$) of FD4^54^. In the Caco-2 model, the $P_{app}$ value peaked between 20 and 40 min, reaching 1.07 × 10^−6^ cm/s, while in the MDCK cell line, the $P_{app}$ value peaked between 40 to 60 min, measuring 1.15 × 10^−6^ cm/s at a concentration of 100 nm (Fig. 4c,d). The $P_{app}$ value in the MDCK cell line appears slightly lower than in the Caco-2 cell line. It should be noted that the MDCK cell model is believed to form more restrictive tight junctions^53,55^ than Caco-2 cells, but here the values are nearly identical. This may be attributed to paracellular permeation occurring through restrictive pores and can be estimated using the Renkin Molecular Sieving Function relating the molecular/hydrodynamic radius (r) to the pore radius (R)^20,21,56^. When (r/R) approaches 1, the overall contribution of the diffusion through the tight junctional pores is limited; thus, FD4 has a larger r that is limiting, and permeability is primarily driven by molecular diffusion. Smaller solutes may have shown some differences. In addition, perturbation of the tight junctions increases the pore radius R, where differences may be observed^20,21,56^. We also determined the $P_{app}$ value of FD4 administered with LAP compared to other established permeability enhancers to increase R transiently, such as Na-caprate (C_10_) and EDTA. Our data show that LAP requires 10,000-fold lower amounts to reach an equivalent $P_{app}$ value of EDTA (1.5 × 10^−6^ cm/s) and 100,000-fold lower to achieve a similar value as Na-caprate. Moreover, at these concentrations required for efficacy, Na-caprate may exhibit toxicity, and EDTA may have a negative effect on calcium and other cation levels available for absorption into the body^57,58^ (Fig. 4e).

Earlier, we showed that the N2-subdomain of LAP has the strongest affinity towards host Hsp60^47^. Thus, we examined its activity as a TJM for FD4 permeability using the Caco-2 model. TEER values of N2 decreased by about 96% compared to the full-length LAP (100%) and 78% for N1, while only 9% for C1 and 30% for C2 (Fig. 4f), indicating that N2 subdomain has a comparable TJ modulatory effect to the full LAP. In the FD4 permeability assay, LAP showed 264% paracellular flux relative to PBS control. In comparison, the N2 (149%) and N1 (133%) showed comparable values, and C1 and C2 led to negligible change (Fig. 4g). A similar FD4 permeability was observed in the presence of a fasted state simulated gastric fluid (FaSSGF) and a fasted state simulated intestinal fluid (FaSSIF) measured after one-hour exposure (Fig. 4h,i). In general, the LAP and N2 subdomain activity was significantly affected by FaSSGF (Fig. 4h), possibly due to the proteolytic activity of the pepsin supplement in the FaSSGF. In contrast, FaSSIF, which contains pancreatin, has no apparent deleterious effect (Fig. 4i), suggesting intestinal conditions may not affect the LAP activity within the tested time window.

Structural analysis revealed amino acid residues from three primary interfaces of LAP that interact with Hsp60, including (i) D101-E108, (ii) S430-Q438, and (iii) K391-I402 (Fig. 2g,h; Fig. 4j). These three synthetic peptides (**Supplementary Table 3**), in combination, promoted FD4 permeability similar to LAP through Caco-2 monolayers, while a random synthetic peptide sequence located outside the LAP-Hsp60 binding domain as control had significantly lower FD4 permeability (Fig. 4k). These data demonstrate peptide mimetics derived from LAP-Hsp60 structural analysis could potentially be used as a TJM for biopharmaceutical delivery.

Analysis of the paracellular flux of bulky molecular model drugs such as FITC-labeled 40 kDa dextran (FD40) or FITC-labeled 70 kDa dextran (FD70) in the Caco-2 cell model showed a similar permeability trend to FD4; however, the paracellular flux of these larger molecules was significantly lower than FD4 (**Supplementary Fig. 5d,e, Supplementary Table 4**). Remarkably, the paracellular flux of FD40 and FD70 was not detected in the MDCK cell line (**Supplementary Fig. 5f**), suggesting that the LAP-mediated paracellular translocation in this cell line is restricted to smaller (<40 kDa) molecules involving the leak pathway.

### LAP amplifies intestinal paracellular transport in mice

**Fig. 5 |:**
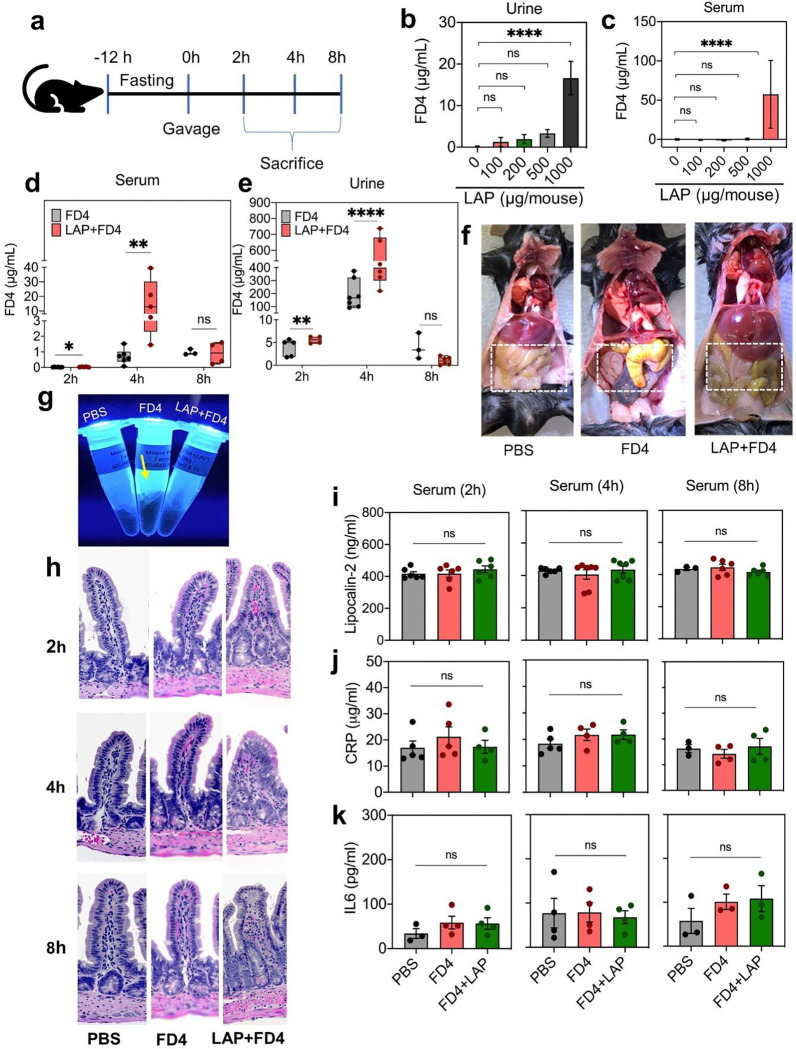
LAP facilitates paracellular permeability across the intestinal barrier in mice **a**. Overview of paracellular permeability study in C57BL/6 mice. **b-c.** LAP-Dose-dependent response for 4-kDa FITC-dextran (FD4) permeability in urine (b) and serum (c) of mice. **d-e.** Time-dependent response for FD4 permeability in urine (d) and serum (e) of mice. **f.** Gross pathology of the intestine 4 h post gavage **g.** Green fluorescence emission from feces (4-h-post gavage from mice treated with FD4 (arrow) but not from LAP+FD4. **h.** Hematoxylin and Eosin (H&E)-stained histopathological images of mouse ileal sections. **i-k.** ELISA analysis of inflammation markers, Lipocalin-2 (**i**) CRP (**j**) and IL-6 (**k**). All error bars represent SEM (n=3–6). ****p < 0.0001; ***p < 0.001; **p < 0.01; ns, no significance.

LAP-mediated intestinal epithelial tight junction modulation and oral macromolecule and biopharmaceutical delivery to blood were also verified in a mouse model. Mice (C57BL/6 mice; 8–12 weeks old; male and female) were fasted for 12 h (with adlib water) and gavaged (200 μl/mouse) with LAP or its subdomain proteins containing FD4, FD40, or peptide drugs. Food was restored before euthanasia using CO_2_-asphyxiation. Initially, we determined the dose effect, where mice received variable concentrations of LAP (100 μg to 1000 μg/mouse, i.e., 5–50 mg/kg body weight) and a constant amount of FD4 (1.5 mg/mouse, i.e., 75 mg/kg body weight) for 4 h (Fig. 5a–c). An increasing concentration of FD4 was detected in urine and serum with increasing concentrations of LAP, showing maximum FD4 level (15 μg/ml) at 1 mg (53 μM) LAP/mouse (Fig. 5b,c, **Supplementary Table 4**).

Subsequently, we determined the LAP-mediated time-dependent translocation of FD4 in the serum by sacrificing mice at 2, 4, and 8 h post-gavage. Mice (n = 5–7 per group) received (200 μl/mouse) LAP (1 mg/mouse i.e., 50 mg/kg body weight) plus FD4 (1.5 mg/mouse, i.e., 75 mg/kg body weight), FD4 alone, or PBS. At 2 h, the FD4 was undetectable in all animals, while at 4 h, the FD4 level was significantly higher (0.3-fold) in animals that received LAP+FD4 compared to the FD4 alone. At 8 h, the FD4 level was significantly low, and the two groups had no differences (Fig. 5d,e).

Examination of the general appearance of the intestine of the 4-h-post gavage group indicated possible accumulation of FD4 (greenish-yellow) residue in the intestine in the FD4-fed group only. In contrast, in the LAP+FD4 group, the intestine appeared normal, as in the PBS-treated group (Fig. 5f). Furthermore, gross examination of the cecum-colon section of mice from all three groups showed no discernible differences or signs of abnormalities (**Supplementary Fig. 6a**). The fecal pellets collected from the colon appeared sausage-shaped with standard consistency. They settled at the bottom of the Eppendorf tubes, indicating the absence of mucus or fluid secretion during LAP exposure (**Supplementary Fig. 6b**).

**Fig. 6 |:**
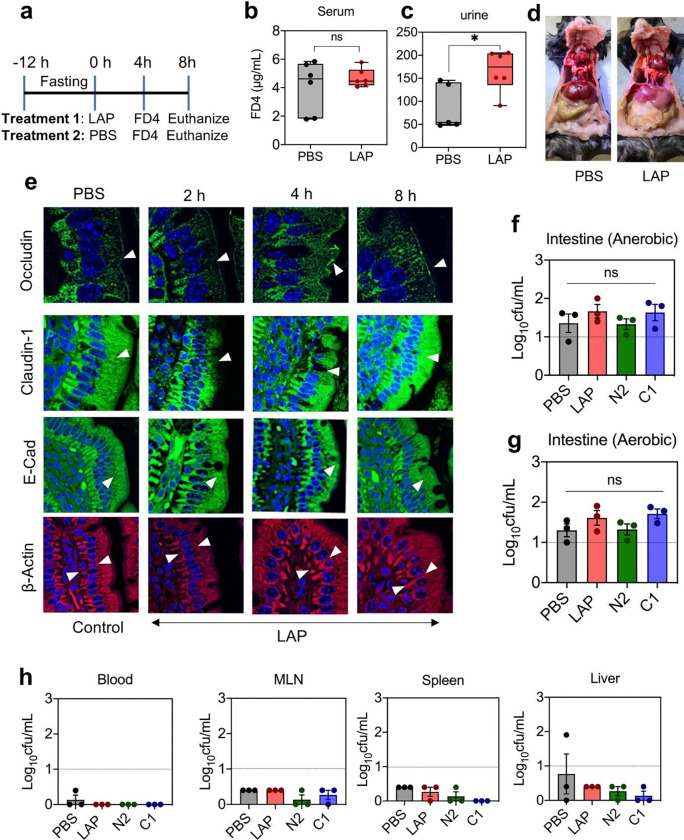
LAP-mediated intestinal epithelial barrier opening is transient **a.** Overview of mice experiment to demonstrate TJ recovery. **b-c.** FD4 permeability in serum (b) and urine (c) of mice after 8 h of LAP treatment. **d.** Appearance of mouse intestine after 8 h of LAP treatment. **e.** Confocal images of ileal tight junction protein occludin, claudin-1, β-actin and E-cadherin after LAP treatment. **f-h.** Intracellular commensal bacterial counts in the intestine after aerobic (**f**) and anaerobic (**g**) incubation, in blood MLN, spleen and liver (**h**) after 4 h of LAP or subdomain proteins treatment. All error bars represent SEM (n = 3–6). ****p < 0.0001; ***p < 0.001; **p < 0.01; ns, no significance.

Interestingly, feces from mice receiving FD4 without LAP emitted fluorescence under UV light; in contrast, the fecal pellet from the FD4+LAP group had faint fluorescence, similar to the PBS-treated control group (Fig. 5g). LAP also facilitated a significant increase in FD40 permeability 4 h postgavage (**Supplementary Fig. 6c,d**). The relative translocation of FD4 was 14-fold higher than FD40 (**Supplementary Fig. 6e**) without affecting the gross pathology or fecal consistency (Fig. 6f,g). These data further indicate that LAP facilitates the paracellular permeation of FD4 across the TJ to enter the bloodstream; however, in the absence of LAP, FD4 primarily accumulates in the intestinal luminal contents, including the feces, which demonstrates it is not absorbed.

Next, we examined if oral administration of LAP would induce any inflammatory response by H&E staining of ileal sections and by analyzing several inflammatory markers in the serum and fecal samples. H&E-stained ileal tissue sections did not show any infiltration of mononuclear or polymorphonuclear (PMN) cells into ileal mucosal or submucosal tissues after 2, 4, or 8 h post gavage (Fig. 5h, **Supplementary Fig. 7a**). Similarly, analysis of inflammatory markers, including lipocalin 2 (LCN2) from both serum and feces and C-reactive protein (CRP), IL-6, and TNFα from serum did not show any significant difference after 2, 4, or 8 h exposure to LAP (Fig. 5i–k, **Supplementary Fig. 7b,c**). Endotoxin (LPS) analysis in the serum sample of mice after 4-h post gavage with LAP did not reveal any difference in LPS levels between LAP-treated and control samples (**Supplementary Fig. 7d**), indicating LAP does not promote translocation of luminal LPS into the bloodstream. Altogether, these data suggest that oral exposure to LAP as a TJM does not cause any detectable inflammatory response in a mouse model. It also suggests that loosening the tight junctional pore radius does not promote the paracellular permeation of compounds much larger than 4 kDa. LPS is heterogeneous and can form aggregates but is generally above 50 kDa^59^.

### LAP-mediated intestinal epithelial barrier opening is transient

To verify the duration of TJ opening in the mouse model, we orally administered LAP (1 mg/mouse) or PBS followed by FD4 (1.5 mg/mouse) after 4 h and euthanized animals at 8 h (Fig. 6a). The FD4 level did not show a significant elevation in serum samples at 8 h (Fig. 6b); however, a marginal increase was noticed in urine (Fig. 6c), indicating the usual lag time for the translocation of FD4 from blood to urine. Nevertheless, the absence of a notable increase in FD4 levels in serum implies TJ recovery and the reinstatement of barrier function. No gross visual changes were observed in the intestine after 8 h of LAP treatment (Fig. 6d).

LAP opens the TJ barrier by promoting cytoskeletal contraction of TJ (claudin, occludin) and AJ (E-cadherin) proteins^30^ and by hijacking caveolar endocytosis of junctional proteins into the endosome^31^. Confocal microscopy of immunostained ileal tissue sections from time-dependent animal experiments (Fig. 6e) did not reveal any significant differences in junctional protein expression at 2 h compared to the control group treated with PBS (Fig. 6e). However, occludin, claudin-1, and E-cadherin mislocalization was pronounced in 4-h post-gavage ileal tissues, indicating epithelial barrier opening (**Supplementary Movie 4**) that agrees with the FD4 translocation data (Fig. 5d,e). At 8 h, the cell-cell junctional protein distribution was restored (Fig. 6e). β-actin, used as a control, was uniformly expressed in all tissue sections.

We also investigated whether LAP-mediated opening of the intestinal epithelial barrier would allow commensal bacteria to translocate to deeper tissues. We collected mouse intestinal sections (Jejunum-ileum; ~10 cm in length) 4 h post-LAP, N2 and C1 (as negative control)-subdomain gavage, treated them with gentamicin for 2 h to kill extracellular bacteria, and plated on BHI agar for intracellular bacterial counts after incubation at aerobic and anaerobic environments. Intestinal intracellular bacterial counts were similar for all treatments (Fig. 6f,g). In addition, blood, mesenteric lymph nodes (MLN), liver, and spleen samples were also analyzed for commensal bacterial burden (Fig. 6h). Our results showed no significant difference in bacterial counts between LAP or subdomain-treated and untreated mice, indicating that LAP treatment does not promote commensal bacterial passage through the intestinal barrier and supporting that changes in the tight junctional pore radius still maintain restrictive barrier integrity (Fig. 6f–h). These findings suggest that the dosage of LAP used in the study can transiently open the TJ barrier, allowing only the intended drugs with molecular weights lower than 4 kDa to pass through without permitting the passage of commensal bacteria into the submucosal layer or deeper tissues.

### LAP augments systemic protein (peptide) drug bioavailability in mice

**Fig. 7 |:**
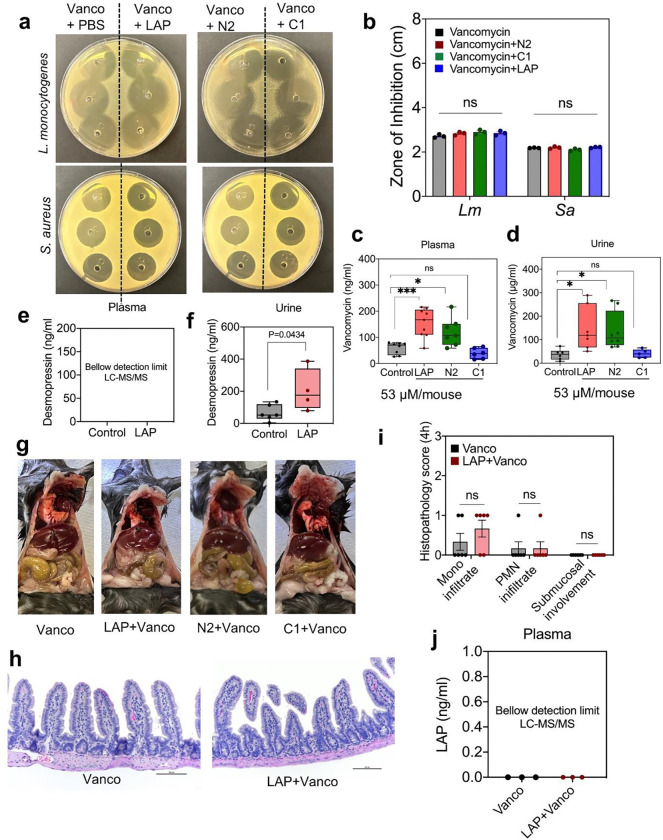
LAP facilitates protein (peptide) drug passage across the intestinal epithelium in mice. **a-b.** Agar diffusion assay showing antimicrobial activity of vancomycin mixed with LAP and subdomain proteins against Lm and Sa (a). Graphical representation of zones of inhibition (b). **c-d.** LAP and subdomain-mediated delivery of vancomycin in serum (c) and urine (d) 4 h post-gavage **e-f.** LAP and subdomain-mediated delivery of desmopressin in plasm (e) and urine (f) 4 h post gavage. **g-i.** Gross pathology (g), Histopathology image (h), and scores (i) **j.** Mass-Spec analysis of LAP in mouse serum following gavage with LAP+vancomycin. All error bars represent SEM (n = 3–8). ****p < 0.0001; ***p < 0.001; **p < 0.01; ns, no significance.

Peptide drugs are used to treat various diseases, but their delivery through the oral route is challenging due to their susceptibility to digestive enzymes and poor bioavailability. Therefore, we examined the bioavailability of two peptide drugs, vancomycin (antibiotic; 1.45 kDa; 100 mg/kg/dose) and desmopressin (vasopressin hormone; 1.13 kDa; 2 μg/kg/dose) after oral gavage in the presence or absence of LAP in a mouse model (**Supplementary Table 5**). LAP (53 μM) was mixed with vancomycin (1.4 mM) or desmopressin (0.17 mM) and orally gavaged into mice. Before the animal experiment, we tested vancomycin’s antimicrobial activity to ensure that mixing LAP with the peptide drug does not interfere with its functionality. We determined the antimicrobial activity of the mixture by analyzing MIC against Lm and *Staphylococcus aureus* (Sa). The MIC of vancomycin with or without LAP was 0.7–1.5 μg for Lm and 1.5–3 μg for *S. aureus* (**Supplementary Fig. 8a**). Antimicrobial activity was also unaffected as tested against the lawn of Lm and Sa cells on brain heart infusion (BHI) agar plates, indicating that LAP does not interfere with the biological activity of vancomycin (Fig. 7a,b).

Mice were gavaged with vancomycin/desmopressin alone or with LAP and euthanized 4 h post-gavage. Drug concentrations in serum and urine were quantified using mass spectrometry. Vancomycin levels in plasma increased by 2.6-fold when co-administered with LAP compared to the control group that only received the vancomycin (Fig. 7c,d). Desmopressin level was below the detection limit in the plasma sample, but its level doubled in the urine (Fig. 7e,f). This is because desmopressin has a plasma half-life of 1–2 h with prolonged (7–10 h) anti-diuretic effects^60,61^, hence it was undetectable in plasma at 4 h and consequently higher accumulation in the urine.

We also examined if the N2-subdomain (~25 kDa) of LAP could facilitate biologics delivery after oral gavage. As shown above, during LAP-Hsp60 interaction, the N domain of LAP forms a complex (Fig. 2). *In vitro* cell culture experiments showed that the N2 subdomain could increase the paracellular permeability of model drug (FD4) delivery across the epithelial barrier (Fig. 4). Hence, we analyzed the ability of the N2 subdomain for vancomycin delivery in the mouse model and compared the results with LAP (positive control) and C1 subdomain (negative control). Our results indicate that co-administering vancomycin orally with N2 significantly improved its oral bioavailability (up to 2-fold), while the C1 subdomain had no effect (Fig. 7c,d). These data demonstrate that the N2 subdomain fragment with a strong affinity towards Hsp60 ^47^ could be used as a TJM for biologics delivery through the intestinal barrier. Examination of gross intestinal appearance (Fig. 7g, **Supplementary Fig. 8b,c**), histopathology scoring (Fig. 7h,i), and fecal consistency (**Supplementary Fig. 8b,c**) showed no tissue inflammation or visible abnormalities.

Concurrently, plasma samples of mice were also analyzed for LAP using mass spectrometry, and LAP was undetectable in the samples (Fig. 7j). This observation suggests that LAP does not translocate to the bloodstream during biologics delivery, which is expected since LAP-Hsp60 forms a complex with IKKβ during NF-κB activation^30^ and presumably remains on the cell surface.

## Discussion

Safe and effective epithelial tight junction modulators are in high demand for improved bioavailability of biologics or medicinal drugs, especially protein/peptide drugs, through the oral route. In the intestine, tight junctions maintain selective absorption with barrier function, enabling rapid adjustments in permeability in response to dietary nutrients or circadian rhythms^62^.

Oral drug delivery technologies exploit such properties to open the junction temporarily for improved drug absorption^20,22–24^. However, TJ modulation must be carefully calibrated to avoid destabilizing critical barrier functions and prevent commensal bacterial translocation or toxin absorption^63,64^. This delicate balance underscores the imperative need for precision in therapeutic approaches. Given their paramount importance, there has been a surge in research to identify modulators that can interact with epithelial junctions without deleterious effects. Therefore, a comprehensive understanding of the specific attributes of intestinal TJ is crucial for developing innovative and effective oral drug delivery systems.

In recent years, microbial virulence factors have been investigated as potential TJ modulators for drug delivery. Pathogenic microbes have evolved intricate mechanisms to manipulate junctional protein complexes to facilitate their invasion and propagation. The mechanism of TJ modulation by pathogens is a dynamic interplay of host-pathogen interactions^65^. The notable specialized virulence proteins that have been investigated include Zot (Zonula occludin toxin) produced by *Vibrio cholerae*^17^, heat-stable enterotoxin (ST) by *E. coli*^66^, EspF by enteropathogenic *E. coli*^67^, and HtrA by *Helicobacter pylori*^68^.

In this study, LAP, a housekeeping metabolic enzyme as a TJM, was investigated since LAP, upon interacting with the host cell receptor, Hsp60, opens the epithelial tight junction barrier by activating MLCK that regulates TJ proteins^30,31,69^. However, the structural interaction between LAP and its receptor, Hsp60, for dynamic interplay leading to downstream signaling events is unknown. LAP is also known as acetaldehyde alcohol dehydrogenase (AdhE), a moonlighting protein with metabolic function^38,70^. It also promotes paracellular translocation of Lm through the intestinal barrier for systemic spread to deeper tissues: mesenteric lymph nodes (MLN), spleen, liver, brain, kidney, lungs, and placenta^30,33,37,41,69,71^. AdhE homologs from *Entamoeba histolytica* and Lm are implicated in pathogenesis, while *E. coli* AdhE, a well-studied enzyme, is not involved in pathogenesis. Applying a housekeeping moonlighting protein such as AdhE/LAP as a TJM^70^, which is not considered a specialized virulence factor, for oral biologics delivery would be an intriguing and advantageous approach.

To better understand the mechanism for LAP-mediated biologics delivery and to enhance our understanding of the molecular mechanisms underpinning Lm pathogenesis, we determined the structure of LAP using cryo-EM (Fig. 1). The LAP structure reveals a mix of oligomers, including dimers, tetramers, and large assemblies with additional flanking C-domains of LAP. Various forms of similar spirosome assemblies have been observed in other homologs of LAP, including AdhE from *E. coli* (**Supplementary Fig. 1** and 4), which has no reported role in pathogenesis^72^. Cryo-EM and computational modeling were used to study the interaction of LAP with Hsp60, indicating the N-domain of LAP interacts along the inner edge of the Hsp60 core, which aligns well with our previous studies. Delineating the LAP-Hsp60 complex (Fig. 2) underscores the functional partitioning within LAP, where the N-terminal domain disassociates and binds to Hsp60. At the same time, the C-terminal domain stays back and binds to InlB for surface anchoring (Fig. 2 and 3). The dissociation of the N-terminal domain is essential for facilitating bacterial detachment from specific sites on the host membrane, thereby enabling the continuation of the infection process and the N-terminal domain after interacting with Hsp60 and IKK-β^30^. Such mobility is essential for the bacteria to navigate through the host’s cellular environment, facilitating the progression of the infection and potentially invading new sites within the host.

The alteration of the intestinal TJ barrier by LAP allows the passage of Lm through the intestinal epithelium and into deeper tissues^30,31^. The TJ modulation mechanism involves changes in TJ proteins’ phosphorylation status, interaction with cytoskeletal proteins, or cellular signaling pathways that influence the assembly or disassembly of TJ complexes^73^. Paracellular transport across tight junctions is facilitated via two distinct routes: the pore and leak pathways^25,73,74^. The pore pathway is characterized by its high capacity and charge-selectivity, allowing the transit of small molecules with diameters of less than approximately 8 Å^21^. In contrast, the leak pathway, which is less capacitive and non-charge selective, permits the passage of larger molecules up to about 100 Å in diameter^75^. When severe epithelial damage occurs, these regulated pathways are overshadowed by the emergence of a tight junction-independent route, referred to as an unrestricted pathway, which is neither size-selective nor charge-selective^73,76^. This unrestricted pathway becomes the predominant mechanism for intestinal permeability, allowing a more expansive and uncontrolled range of molecules to traverse the epithelial barrier^73^.

Typically, 4-kDa dextran molecules are confined to the leak and unrestricted pathways, while 70 kDa dextran molecules are restricted solely to the unrestricted pathway^75^. However, in our study, we observed that while 4-kDa dextran could traverse, there was a significant decrease in the paracellular flux of 40 and 70 kDa dextran in Caco-2 cells and no flux at all in MDCK cells (Fig. 4). In the mouse model, both 4 kDa and 40 kDa molecules can traverse the gut barrier (Fig. 5–7); however, levels of 40 kDa are significantly lower than the 4 kDa (**Supplementary Fig. 6e**), suggesting that the leak pathway is the predominant route for LAP-mediated paracellular transport. Furthermore, the LAP-mediated leak pathway is dose-dependent and is facilitated via binding of N domain to epithelial Hsp60, while the C-terminal domain helps LAP to remain anchored on the bacterial cell surface^37^. The functional assays also substantiate our structural complex model, reinforcing the association between the host receptor Hsp60 and the N-terminal domain of LAP critical for epithelial barrier opening and medicinal peptide drug delivery.

The engagement of LAP with epithelial Hsp60 is essential for enabling the translocation of Lm across the TJ barrier^41^ by activating the NF-κB and MLCK signaling pathways^30,31^. The relevance of this pathway is also substantiated in the MLCK knockout mouse (MLCK^−/−^) model, in which serum vancomycin levels remained unchanged in the presence or absence of LAP (**Supplementary Fig. 9**).

During Lm infection, LAP interaction with its cognate receptor, Hsp60, activates NF-κB to induce an inflammatory response marked by increased TNFα and IL-6 secretion^30^. Remarkably, purified LAP did not show any adverse inflammatory response as the levels of the inflammatory markers (CRP, LCN2, TNFα, IL-6) remained unchanged. Furthermore, the LAP-mediated opening of the TJ was transient, showing a maximum delivery of drugs four hours post gavage and restoration of cell-cell junctional proteins (occludin, claudin-1, and E-cadherin) by 8 h. Luminal commensal bacteria or LPS translocation in the submucosal layer or the extra-intestinal compartments (blood, MLN, liver, spleen) were not observed, suggesting LAP as a TJM is safe without exhibiting any potential adverse effects on health.

In conclusion, this study comprehensively analyzes the LAP (AdhE) structure and its interaction with Hsp60 and InlB, revealing its intricate molecular architecture as a tight junction modulator and its role in bacterial pathogenesis. Cryo-EM shows LAP as dimers and tetramers, with the tetrameric form displaying a spirosome-like assembly. The model for the fully assembled InlB-LAP-Hsp60 complex provides comprehensive insights into the mechanism of Lm pathogenesis through the LAP-dependent pathway established in our previous studies^30,31,37,38,41^. This study further confirms that LAP is a promising paracellular permeability enhancer that temporarily opens the TJ barrier via the leak pathway, allowing only the intended molecules to pass through without permitting the transfer of commensal microflora or their components. Furthermore, peptide mimetics derived from LAP facilitated drug analog delivery through the epithelial barrier *in vitro,* which is encouraging. Overall, these findings have important implications for oral drug delivery and patient compliance, as LAP may enable the delivery of drugs and biologics that would otherwise have poor oral bioavailability.

## Methods

### Mice

Eight to twelve-week-old, male or female, C57BL/6 mice bred in Hansen Animal Facility, Purdue University, were used for the study. Mice were housed in climate-controlled environments and provided ad libitum feed and water. Food was removed from the cages twelve hours prior to the experiment to ensure more uniform drug absorption, preventing any interference of the test-drug mixing with food^77^. Mice were administered 200 μL of drug/ analog with LAP or subdomain protein suspension orally using a stainless-steel ball-end feeding needle (Cadence Science). The control mice received either vehicle or vehicle plus the drug. The food was returned after oral administration, and the mice were sacrificed after 2, 4 or 8 h using CO_2_ asphyxiation. All animal procedures (IACUC Protocol no. 1201000595) were approved by the Purdue University Animal Care and Use Committee, which adheres to the recommendations of the Guide for the Care and Use of Laboratory Animals published by the National Institute of Health.

### Cell Lines

The human colon carcinoma Caco-2 cell line (ATTC # HTB-37) and Madin-Darby canine kidney (MDCK, ATTC # CCL-34) cell line from 25–35 passages were cultured in Dulbecco’s Modified Eagle’s medium (DMEM) (Thermo Fisher Scientific) supplemented with 4 mM L-glutamine, 1 mM sodium pyruvate and 10% heat-inactivated fetal bovine serum (FBS; Atlanta Biologicals). All cell lines were maintained at 37°C with 5% CO_2_.

### Expression and purification of LAP and Hsp60

The full-length LAP gene (2.6 kb) from Lm was cloned into a pHis-*II* vector using NcoI and XhoI restriction sites, for expression with an N-terminal 6-His tag, including a tobacco etch virus (TEV) protease cleavage site (ENLYFQ|G). LAP was expressed in *E. coli* BL21 (DE3) competent cells (NEB) and grown in 2XYT media with 50 μg/mL followed by induction with 0.2 mM IPTG at 20°C for 4 h until the OD_600_ reached 1.0. Cells were harvested by centrifugation at 4,553× g for 10 min. The cell pellets were resuspended in a buffer containing 1X PBS, pH 7.4, and protease inhibitor (0.2 mM) and DNase I (10 μg/mL). The samples were then lysed in a high-pressure homogenizer (Avestin Emulsiflex C3). The cell lysates were clarified by centrifugation at 39,204×g for 20 min, and supernatants were incubated with Ni-NTA agarose beads (Qiagen). The Ni-NTA beads were washed with buffer A containing 50 mM imidazole, and bound protein was eluted with buffer A containing 500 mM imidazole. The N-terminal His-tag was cleaved by TEV protease during an overnight dialysis step against buffer A, containing 1X PBS, pH 7.4, 1 mM DTT, and 0.5 mM EDTA at 4°C. The samples were further purified by gel filtration (Superdex 200 increase, GE Healthcare) in buffer A (1X PBS, pH 7.4). The peak fractions were pooled and concentrated with Amicon Ultra 50,000 MWCO centrifugal filters (Millipore) up to 10 mg/ml. LAP subdomains were purified similarly to full-length LAP.

Purified human recombinant Hsp60 from a vendor (Cayman) was mixed with the LAP at a 2:1 (w/w) ratio and incubated for 4 h at 4°C.

### Negative staining of protein preparation by electron microscopy

For negative staining, 3 μl of purified LAP (0.01 mg/ml) were applied to glow-discharged carbon coated Cu (400 mesh) grids and incubated for 1 min. The grids were washed twice with water and were incubated with 0.5% (w/v) uranyl acetate for 20 sec for negative staining. Excess uranyl acetate was blotted away, and the grids were dried. The grids were then imaged using a Tecnai T20 transmission electron microscopy (TEM; FEI) operated at 200 kV. Images were recorded using a CCD camera (Gatan US1000 2 k × 2k) with a defocus set to 1.5 μm, corresponding to a pixel size of 2.4 Å.

### SDS-PAGE and Western blotting

In the SDS-PAGE procedure, protein samples mixed with sample buffer were heated and loaded onto a 10% polyacrylamide gel for electrophoresis. Following electrophoresis, proteins were transferred onto a polyvinylidene difluoride (PVDF) membrane using a wet transfer system. The membranes were then blocked with nonfat dry milk (5% w/v) to prevent non-specific binding and incubated with mouse monoclonal antibodies against LAP and single chain antibodies to N1, N2, C1, and C2 domains and secondary antibodies (**Supplementary Table 6**) described by Liu et al^37^, specific to the target protein. Finally, the protein bands were visualized using Enhanced Chemiluminescence.

### Cryo-EM sample preparation and image processing

For LAP alone, 3 μl of purified LAP (1.2 mg/ml) were applied to glow-discharged QUANTRIFOILR1.2/1.3 grids (400 mesh) with a home-coated gold layer. For the LAP-Hsp60 complex, 3 μl of purified LAP with Hsp60 (2:1 molar ratio) was applied to glow-discharged QUANTIFOIL R 1.2/1.3 (200 mesh) grids. The grids were blotted in 100% humidity and plunge-frozen using a Vitrobot Mark IV (ThermoFisher Scientific). Movies were collected on a Titan Krios microscope (ThermoFisher) operated at 300 kV with a nominal magnification of 81,000x using a K3 direct electron detector (Gatan) operated in super-resolution counting mode using Leginon^78^ or EPU (ThermoFisher).

For the LAP-Hsp60 dataset, images were recorded at a defocus range of 1.5 to 2.5 μm, with a calibrated physical pixel size of 0.527 Å/pixel. With 40 frames recorded, the total exposure time was 3.12 s, leading to a total dose rate of 57.8 e^−^/Å^2^. A total of 5,317 movies were collected and motion correction and image processing performed using cryoSPARC^79^. A total of 2,645,207 initial particles were extracted using template picking. After multiple rounds of ab initio modeling and heterogeneous refinement, multiple 3D classes were observed for the LAP dimer, LAP tetramer, and LAP-Hsp60. Each 3D class was further refined using iterative rounds of 2D classification, non-uniform refinement, and local refinement. For the final LAP dimer reconstruction, 77,918 particles were used in C2 symmetry, resulting in a 3.19 Å resolution map. For the final LAP-Hsp60 reconstruction, 73,657 particles were used in C1 symmetry, resulting in a 3.94 Å resolution map. Another refinement was performed using C7 symmetry to produce a 3.37 Å resolution map of Hsp60 alone. The LAP tetramer from this dataset was almost identical to that observed in our LAP-only dataset above; however, to lower resolution, that is not reported.

Initial models of LAP and the LAP-Hsp60 complexes were prepared using AlphaFold^49^. All manual model building was performed using COOT^80^, and real space refinement was performed for all structures using PHENIX^81^. Data collection and refinement parameters for the reported cryo-EM structures in this study are summarized in **Supplementary Table 1**.

### Computational modeling and docking

We modeled complexes of (1) LAP with Hsp60 and (2) LAP with InlB using AlphaFold/HDOCK^49,50^ and AlphaFold/LZerD^49,51^, respectively. For the LAP-Hsp60 complex, AlphaFold was first used to model the complex between the N-domain of LAP and Hsp60. While the initial results agreed with the cryo-EM maps, inter-residue contacts were not resolved. Therefore, this was used as a starting model and HDOCK was used for subsequent docking to produce the final set of docked models. For the LAP-InlB complex, AlphaFold was used to prepare a model of InlB, which was used with the LAP structure for docking studies using LZerD. These subcomplexes were used to form a model for the complete LAP-InlB-Hsp60 intercellular spanning complex.

### Epithelial permeability assay for drug analogs

Caco-2 and MDCK cells were grown as monolayers on Transwell inserts with 3.0 μm pores (Corning-Costar) for 21–28 days. TEER was measured to monitor the monolayer integrity (Millicells Voltmeter, Millipore). A TEER value of at least 300U/cm^2^(±10) was used as the basal value to monitor the monolayer integrity^41^. Purified LAP and subunit proteins (100 nM) were added to the apical side of the Transwell system, with a marker molecule FITC-labeled dextran of size 4, 40 and 70 kDa (2 mg/ml, Sigma-Aldrich) and after 1 h incubation at 37°C in 5% CO_2_, the liquid was collected from the basal well, and fluorescence from FITC was measured (Em: 485 nm; Ex: 520 nm; Spectramax, Molecular Devices). In addition, change in TEER value was measured for each treatment^30^. $P_{app}$ was calculated using the following formula:

$$P_{app}=\frac{dQ}{dt}/\left(A\cdot C_{0}\right)$$

Where:

$\frac{dQ}{dt}$ is the rate of appearance of the drug (FD4) on the basolateral side of the membrane,

A is the surface area of the membrane,

$C_{o}$ is the initial concentration of the drug on the apical side.

The $P_{app}$ of FD4 in the presence of LAP was determined at various time intervals ranging from 0 to 60 min, encompassing a range of LAP dosages. Additionally, a comparative analysis was done to assess the $P_{app}$ of FD4 in the presence of Na-Caprate (10 mM), EDTA (1 mM), and LAP (100 nM), utilizing the equation mentioned above.

During *in vitro* epithelial permeability assay, FaSSGF and FaSSIF were prepared as before^82^ with minor modifications. For FaSSGF, KCl, KH_2_PO_4_, NaHCO_3_, NaCl, MgCl_2_·6H_2_O, and (NH_4_)_2_CO_3_ were mixed, followed by the addition of pepsin, CaCl_2_, and water, with the pH adjusted to 1.5 using HCl. For FaSSIF, the same salts were combined, then pancreatin, CaCl_2_, and water were added, adjusting the pH to 7.0 with NaHCO_3_. Both fluids were stored at 4°C until use.

### Peptide mimetics synthesis and epithelial permeability testing

Structural analysis of LAP-Hsp60 followed by computational docking experiment revealed amino acid residues of the N-domain of LAP that interact with Hsp60. To further verify if these Hsp60 interacting primary residues (Fig. 2h, 4j) would be able to increase epithelial barrier permeability, we synthesized three peptides: (i) Peptide 1 (N99-E108: VINEDVQTGVIE), Peptide 2 (K391-I402: KAFGIRMKACRI), Peptide 3 (S430-N44: **K**SYGKNSVSQNVSATN with extra K residue in the N-terminal end for improved water solubility) (NovoPep, Shanghai, China) (**Supplementary Table 6**). In addition, we also synthesized another peptide (Peptide 4, E630…D639: DKENNIKYPLAD), which is located outside the LAP-Hsp60 interacting domain and used as a negative control. All peptides were purified by HPLC (Schimadzu LCMS-2020) to achieve a purity of >98%, and 14 mg of each peptide was received from NovoPep (**Supplementary Table 3**).

To test the activity of peptides, we used the transwell assay as above. Briefly, Caco-2 cell monolayers on Transwell inserts (Corning-Costar) (n=4–8) showing a TEER value of about 500U/cm2(±10) were inoculated with peptide preparations. Peptides 1, 2, and 3 each at 80 μg/ml (total 240 μg/ml) and Peptide 4 at 80 μg/ml were dissolved in HBSS without phenol red indicator (Gibco). [Note: peptide concentrations in the 12–17 μg/ml range did not respond satisfactorily.] LAP (1 mg/ml) and EDTA (1 mM) were dissolved in HBSS and added to the apical side of the Transwell system, with FD4 (2 mg/ml, Sigma-Aldrich) and after 1 h incubation at 37°C in 5% CO_2_, liquid was collected from the basal well, and fluorescence was measured (Em: 485 nm; Ex: 520 nm; Spectramax, Molecular Devices).

### Biologics delivery through oral route in a mouse model

Two types of biologics were tested in this experiment: (1) a drug analog, non-metabolizable FITC-labeled 4 or 40 kDa dextran (FD4, FD40) and (2) peptide drugs, vancomycin and desmopressin.

FD4 or FD40 (15 mg/100 μl PBS, Sigma-Aldrich) were dissolved in PBS with or without LAP (1 mg/mouse), and mice were orally gavaged with 200 μl/mouse at 2, 4 or 8 h before sacrifice by CO_2_ asphyxiation. Serum and urine (voluntary discharge during euthanasia or directly from the bladder) samples were collected and diluted in PBS, and fluorescence was measured (Em: 485 nm; Ex: 520 nm; Spectramax, Molecular Devices). The FD4/FD40 concentration was calculated using a standard curve^30^. The serum and urine from the naïve mice were used as the background control.

Vancomycin (1.45 kDa; 100 mg/kg/dose) and desmopressin (1.13 kDa; 2 μg/kg/dose) were dissolved in PBS (pH 8.1) containing Tween 20 (0.2%; v/v) and prepared with or without LAP or subdomain proteins (1 mg/mouse). Mice were orally gavaged with 200 μl/mouse four hours before sacrifice by CO_2_ asphyxiation. Plasma and urine samples were collected, and drug concentrations were calculated following LC/MS/MS (Agilent 6460 QQQ) analysis at the Purdue University metabolomic profiling facility.

### Gross pathology

During the mouse dissection, we meticulously examined intestinal organs and tissues, including the cecum and colon, for fluid accumulation, fecal consistency, and gross pathology. Additionally, fecal samples were analyzed under UV light (280 nm) to visualize the FD4 presence in the sample.

### Histopathology

Thin tissue sections (5 μm) from mice samples were stained with hematoxylin and eosin, and a board-certified veterinary pathologist microscopically examined the slides and the interpretations were based on standard histopathological parameters. The pathologist, who was blinded to the treatment groups, compared the ileal sections to the controls. To determine the extent of the inflammation, the mouse ileal tissues were scored on a scale of 0–3 for three parameters, yielding a maximum score of 1. The scoring parameters were the amount of polymorphonuclear leukocyte infiltrate, mononuclear infiltrate and involvement of the submucosa. To grade the amount of polymorphonuclear leukocyte infiltrate and mononuclear infiltrate, the following histomorphological scale was used: 3 = markedly increased, 2 = moderately increased, 1 = slightly increased and 0 = normal. To grade the involvement of the submucosa the following histomorphological scale was used: 3 = 50% or greater of the submucosal diameter, 2 = 10%–50%, 1 = < 10% and 0 = normal.

### Immunofluorescence staining and confocal microscopy

The mouse ileal tissue sections were fixed with 10% formalin and embedded in paraffin. The tissues were sectioned (5-μm thick), deparaffinized, and rehydrated for antigen retrieval by immersing the slides in boiling sodium citrate buffer (10 mM, pH 6.0) or 0.01 M Tris/EDTA (pH 9.0), for 10 min. The tissue sections were permeabilized and blocked with PBS containing 0.3% Triton X-100 (Sigma-Aldrich) and 3% normal goat serum (Cell signaling) and immunostained with specific primary antibodies (**Supplementary Table 6**) by incubating overnight at 4°C^30,69^. Primary antibodies included antibodies to claudin-1 (1:200 dilution), occludin (1:150 dilution), E-cadherin (1:200 dilution), ZO-1 (1:100 dilution), and Phailloidin that binds to β actin (1:200 dilution). Slides were then rinsed with PBS (three cycles, 5 min), and were incubated with respective Alexa Fluor 488/555-conjugated secondary antibody (1:500 dilution) for 2 h at room temperature followed by 3× washing with PBS (5 min each). The nuclei were stained with DAPI (500 ng/mL; Cell signaling), and slides were mounted in ProLong antifade reagent (Invitrogen).

For antibody labeling in cell lines, Caco-2 cells were cultured until they reached 40–50% confluence on eight-well chambered slides (Millipore). After the treatment, the cells were stabilized with a 3.7% formaldehyde solution in PBS for 20 min. The cells were permeabilized and blocked using a PBS mixture containing 0.3% Triton X-100 and 3% BSA (Sigma-Aldrich), and incubated for 1 h at ambient temperature. They were then exposed to primary antibodies (**Supplementary Table 6**), including antibodies to LAP at a 1:50 ratio, ZO-1 at a 1:100 ratio, and Hsp60 pAb also at a 1:100 ratio, or a blend of these primary antibodies in the dilutions mentioned and incubated overnight at 4°C. Subsequently, the cells underwent three 5-minute washing cycles with PBS and were treated with corresponding Alexa Fluor 488/555/647-tagged secondary antibodies at a 1:500 dilution for 2 h at room temperature. Finally, cell nuclei were stained using DAPI at a concentration of 500 ng/ml (sourced from Cell Signaling), and slides were preserved using ProLong antifade reagent from Invitrogen.

All images were acquired using a Nikon A1R MP confocal microscope^30^. The X-Z and Y-Z cross-sections were produced by orthogonal reconstructions from z-stack scanning at 0.15-μm intervals taken with ×60 oil immersion objective in a 5-μm-thick paraffin-embedded tissue section or Caco-2 monolayers. Three-dimensional reconstructions were performed using Nikon NIS Elements software (Nikon Instruments Inc.)/ Zeiss Zen lite software. Post-acquisition processing was done in FIJI software.

### Inflammation markers and ELISA

Levels of Lipocalin-2 was quantified from mouse fecal and serum samples while CRP, IL-6, TNF-α and LPS were analyzed in mice serum samples as before^30,69,83^. Lipocalin-2, CRP, IL-6 and TNF-α concentrations were determined using high-sensitivity sandwich ELISA kits (R&D system and Millipore Sigma) as per the manufacturer’s instruction. At the same time, LPS levels are measured through the Limulus Amebocyte Lysate (LAL) assay (Thermo Fisher Scientific), leveraging the clotting response of horseshoe crab blood to detect endotoxins.

### Bacterial invasion of mouse tissues and organs

To enumerate invasive bacterial counts in tissues, the ileal tissue sections (~10 cm) were first flushed, washed three times with sterile PBS, and suspended in 5 mL of RPMI-5 containing 50 mg/ml gentamicin^30^. The cell suspensions were incubated at 37°C with 5% CO_2_ for 2 h to kill any extracellular bacteria. The tissue samples were then homogenized using a tissue homogenizer in 5 mL PBS. To enumerate commensal bacteria, the samples were serially diluted in PBS and plated onto Brain Heart Infusion agar plates (BHI, RPI) incubated aerobically or in a candle jar under an oxygen-deprived environment. Bacterial counts were also monitored in blood, MLN, liver, and spleen as before^30^.

### Quantification and statistical analysis

The experimental data were analyzed using GraphPad Prism software v8 (La Jolla, CA). The unpaired Student’s t-test was used in experiments involving comparisons between two datasets. In cases where comparisons were made between more than two datasets, either a one-way or two-way analysis of variance (ANOVA) followed by Tukey’s multiple-comparison test was conducted. Statistical significance for mouse microbial counts was determined using the Mann-Whitney test. All data presented are representative of a minimum of 3 independent experiments, and the specific number of mice per group is indicated in the corresponding figure legends. Unless stated otherwise, the data from all experiments are expressed as the mean ± standard error of the mean (SEM).

## Highlights

- Cryo-EM shows LAP forms multimers with the N-domain interacting with Hsp60.
- LAP enhances the oral bioavailability of peptide drugs, outperforming other enhancers at lower doses.
- LAP increases intestinal drug permeability in mice without any inflammatory response.
- LAP-mediated TJ modulation is reversible without permitting luminal bacterial passage.
